# Sirolimus Enhances Cyclosporine A-Induced Cytotoxicity in Human Renal Glomerular Mesangial Cells

**DOI:** 10.1155/2012/980910

**Published:** 2012-01-23

**Authors:** Séin O'Connell, Craig Slattery, Michael P. Ryan, Tara McMorrow

**Affiliations:** Renal Disease Research Group, UCD School of Biomolecular and Biomedical Research, UCD Conway Institute, University College Dublin, Dublin, Ireland

## Abstract

End Stage Renal Disease (ESRD) is an ever increasing problem worldwide. However the mechanisms underlying disease progression are not fully elucidated. This work addressed nephrotoxicity induced by the immunosuppressive agents' cyclosporine A (CsA) and sirolimus (SRL). Nephrotoxicity is the major limiting factor in long term use of CsA. SRL causes less nephrotoxicity than CsA. Therefore investigations into the differential effects of these agents may identify potential mechanisms of nephrotoxicity and means to prevent ESRD induced by therapeutic drugs. Using ELISA, Western blotting, quantitative PCR and a reporter gene assay we detailed the differential effects of CsA and SRL in human renal mesangial cells. CsA treatment increased profibrotic TGF-**β**1 secretion in human mesangial cells whereas SRL did not, indicating a role for TGF-**β** in CsA toxicity. However we observed a synergistic nephrotoxic effect when CsA and SRL were co-administered. These synergistic alterations may have been due to an increase in CTGF which was not evident when the immunosuppressive drugs were used alone. The CsA/SRL combination therapy significantly enhanced Smad signalling and altered the extracellular matrix regulator matrix metalloproteinase 9 (MMP-9). Inhibition of the ERK 1/2 pathway, attenuated these CsA/SRL induced alterations indicating a potentially significant role for this pathway.

## 1. Introduction

Cyclosporine A has improved allograft survival and the quality of life for solid-organ transplant recipients [1]. Its effectiveness in transplantation by suppression of the immune system has led to its use in treating autoimmune diseases [2]. CsA inhibits the immune system by binding to cyclophilin, this complex then inhibits calcineurin, which in turn inhibits the translocation of the nuclear factor of activated T cells (NFAT) and subsequent gene transcription [3]. Calcineurin inhibitors (CNI) can cause nephrotoxicity involving acute renal vasoconstriction progressing on to glomerulosclerosis, tubulointerstitial fibrosis, and renal failure.

 Due to CNI-induced nephrotoxicity, the use of the immunosuppressive agent sirolimus (SRL) in transplantations is becoming more widespread. SRL has a different mechanism of immunosuppression compared to CNIs, and a lesser degree of nephrotoxicity is observed with SRL compared to that observed with the calcineurin inhibitors [4, 5]. When administered with a CNI, SRL may prevent the observed nephrotoxic effects. In both animal and human studies, SRL has been suggested to have a protective role when administered in conjunction with CsA [6, 7]. The Rapamune US Study Group conducted a large multicentre clinical trial in which the efficacy of SRL compared to azathioprine for reducing acute renal allograft rejection was investigated. The use of SRL reduced occurrence and the severity of biopsy-proven acute rejection episodes [8]. However, there have also been studies indicating enhanced nephrotoxicity when SRL and CsA are used in combination [9, 10]. In another study, the authors showed that a combination of SRL and CsA significantly potentiated the nephrotoxic actions of CsA by augmenting transforming growth factor *β* (TGF-*β*) [11].

TGF-*β* has been implicated as a major factor in the development of chronic CNI toxicity. Increased TGF-*β* levels have been observed in renal cells exposed to CsA, in animal models of CsA toxicity and in patients with CNI nephropathy [12–15]; however, the nephrotoxicity caused by CsA remains to be fully elucidated. In this study, the role of the glomerulus in *in vitro *CsA toxicity was investigated. Our hypothesis was to determine whether CsA or SRL had direct detrimental effects on the glomerulus and identify the possible mechanisms involved, using glomerular mesangial cells.

Human mesangial cells (HMCs) are key cells of the glomerulus and have an important role in regulating glomerular structure and function and have the potential to contribute to glomerulosclerosis by secreting profibrotic mediators, which can alter extracellular matrix (ECM) balance and disrupt renal function [16]. This may be an important mechanism in renal disease progression and the upstream signalling pathways involved in CsA-induced renal dysfunction are not well characterised and warrants further investigation. One intracellular pathway that may potentially be involved in immunosuppressive-induced renal damage is that of the mitogen-activated protein kinase (MAPK) family, which has been implicated in TGF-*β*-induced cytotoxicity [17]. The analysis of the differential activation of this pathway may provide a novel insight into the mechanisms of nephrotoxicity caused by CsA.

## 2. Methods

### 2.1. Cell Culture and Treatment

Human mesangial cells (HMCs) were used during the course of this work [18]. The HMCs were grown in RPMI 1640 containing 5% FCS, penicillin, streptomycin, and L-glutamine and maintained at 37°C in a humidified atmosphere containing 95% air, 5% CO_2_. CsA was obtained from Sigma-Aldrich (Cat no. C1832), and SRL was obtained from Merck Biosciences (Cat no. 342500). Confluent HMCs were treated with CsA, SRL, or CsA/SRL cotreatment. Cells used in the inhibitor studies were pretreated for 1 hour with UO126, prior to incubation with each drug treatment.

### 2.2. Cell Morphology and Viability

HMC morphology was analysed using phase contrast microscopy. Mesangial cell viability was assessed using the resazurin conversion (Sigma-Aldrich, 7017) cell viability assay. This assay was conducted according to the manufacturer's protocol. The viability of the cells was expressed as a percentage of the absorbance recorded for control cells.

### 2.3. Quantitative Polymerase Chain Reaction (PCR)

Total RNA was isolated using the trizol method from HMCs, according to the manufacturer's protocol (Sigma-Aldrich, T9424). 1 *μ*g of total RNA was used to synthesis cDNA. A Real-Time PCR TaqMan assay was used to quantify the relative expression levels of genes of interest and has been described previously [13]. Briefly, cDNA was amplified on the ABI 7900HT Sequence Detection System at default thermal cycling conditions: 2 min at 50°C, 10 min at 95°C for enzyme activation, and then 40 cycles of 15 sec at 95°C for denaturation and 1 min at 60°C for annealing and extension. Results were analysed using the delta Ct method of analysis. Applied Biosystems commercial assays were used for all genes of interest, TGF-*β* (Hs99999918_m1), CTGF (Hs00170014_m1), and MMP-9 (Hs00234579_m1). The ribosomal 18S gene was used as an endogenous control for normalisation of the target genes. A negative control containing all reaction components except for Superscript for each set of samples was used.

### 2.4. TGF-*β* ELISA

A TGF-*β*1 ELISA was used to determine the effect CsA had on secreted TGF-*β*1 protein levels in the media of HMCs. This was done according to the manufacturing company's (Cat no. DB100B, R&D systems) protocol. The specificity and sensitivity of the assay was assessed using 5 ng of TGF-*β*1 as a positive control and sterile water as a negative control.

### 2.5. TGF-*β* Smad Responsive Reporter Gene Assay

Transfection reagent-DNA complex was prepared by adding serum-free medium to a sterile eppendorf tube. FuGene (Roche) reagent was added directly to the tube and then 1 *μ*g of TGF-*β* Smad responsive (CAGA) luciferase reporter DNA plasmid (a gift from Dr. Roel Goldschmeding) and 1 *μ*g of control plasmid (renilla) (Promega). For multiple-well addition, components were scaled up proportionally. Cells were transfected overnight at 37°C. Following transfection, cells were treated with each immunosuppressive agent for the required time period. Cells were lysed using passive lysis buffer (Promega) and put into fresh tubes. The luminometer was programmed to perform a 2-second premeasurement delay followed by a 10-second measurement. Equal volumes of cell lysate and Luciferase reagent were added together and the luminescence reading recorded. Stop and glo reagent was added to halt the reaction and another reading performed. This process was repeated for all samples. Control lysates were made from untransfected cells to determine background luminescence levels and a positive control of cells treated with 5 ng TGF-*β* was used.

### 2.6. Western Blot Assay

Total protein was isolated from HMCs using the RIPA buffer method (Sigma-Alridch, R0278) according to the manufacturer's protocol. The SDS-PAGE procedure used was that of Laemmli [19]. Expression levels of renal proteins following CsA treatment was determined by Western blot and has been described previously [15, 20, 21]. Proteins of interest were detected using the following antibodies according to the manufacturer's protocol (rabbit anti-ERK 1/2, Cell Signalling Technology, 9211S and 9211). Time-matched controls were used in the phosphorylation studies.

### 2.7. Statistical Analysis

Statistical analyses were performed using GraphPad Prism 4.0. Data was analysed by one-way analysis of variance (ANOVA), and multiple comparisons between control and treatment groups were made using the Bonferroni posttest. A student *t*-test was used for assessing statistical differences between two groups. A probability of 0.05 or less was deemed statistically significant. Results were expressed as the mean ± SEM. The following scheme was used throughout the work; *P < 0.05, **P < 0.01, ***P < 0.001.

## 3. Results

### 3.1. CsA/SRL Caused Synergistic Nephrotoxicity in HMCs

**Figure 1:**
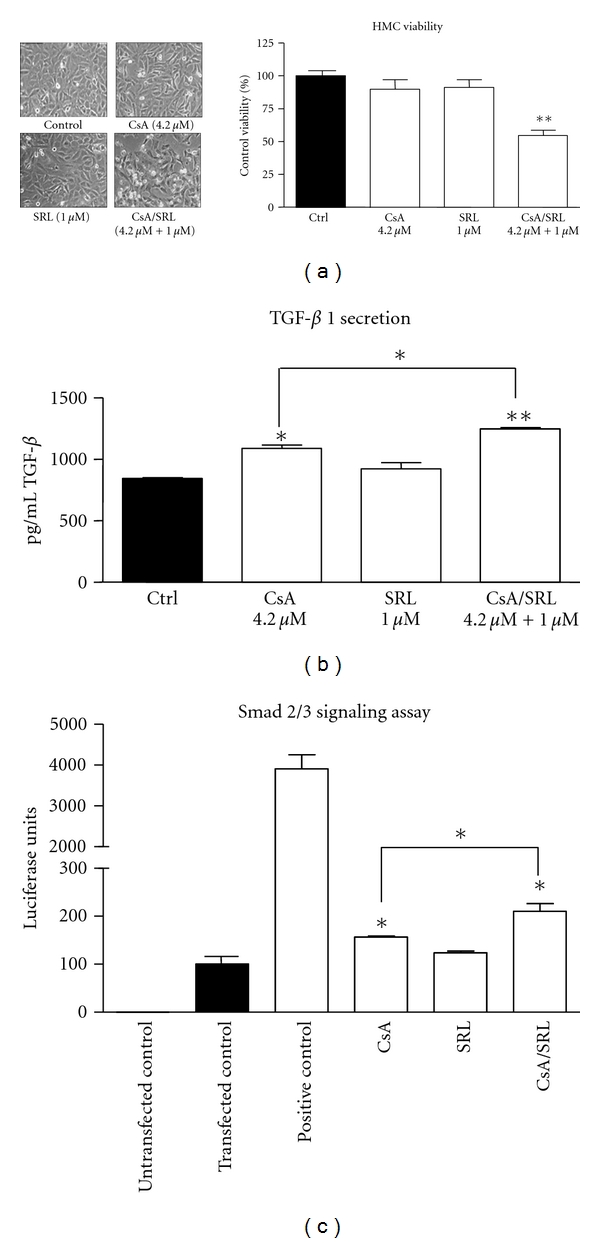
CsA/SRL caused synergistic nephrotoxicity by increasing TGF-*β* and smad signalling in HMCs. HMCs were grown to confluency and treated with vehicle, 4.2 *μ*M CsA, 1 *μ*M SRL or CsA/SRL, 4.2 *μ*M CsA + 1 *μ*M SRL for 24 hours. (a) Phase contrast micrographs were taken using a CCD camera mounted on a Nikon microscope. Magnification 200X. Cell viability was measured using the resazurin conversion assay. (b) 24-hour TGF-*β*1 secretion was detected using a TGF-*β*1 ELISA. (c) Smad activation was measured using a Smad responsive luciferase construct. Smad responsive luciferase values were normalised to a control renilla luciferase construct. Each column represents the mean ± SEM of a minimum of 3 independent experiments performed in duplicate. Data was analysed by ANOVA and comparisons between control and multiple treatment groups were made using the Dunnet posttest. *Indicates statistical difference compared to control. *P < 0.05, **P < 0.01.

Microscopic examination highlighted that treatment with CsA or SRL alone for 24 hours had no significant effect on HMC morphology (Figure 1(a)). However, 24-hour treatment with CsA/SRL combination did cause alterations in HMC morphology (Figure 1(a)). An elongation of normal HMC shape accompanied with gaps in the cell monolayer and cell death (indicated by round floating cells) was observed. Treatment for 24 hour with CsA or SRL alone had no significant effect on HMC viability. Treatment with CsA/SRL drastically reduced HMC viability (100 versus 54 ± 3.9, *P* < 0.05). The concentrations of CsA and SRL used in this study were chosen following dose response studies, and it approximates to concentrations used *in vivo* and reflect concentrations in the kidney.

Treatment with CsA for 24 hours caused a significant increase in TGF-*β*1 secretion (843.1 ± 7.1 versus 1089.5 ± 26.1 pg/mL; *P* < 0.05) as demonstrated by ELISA (Figure 1(b)). SRL alone did not cause any significant alterations in TGF-*β*1 secretion following 24-hour administration. However, cotreatment of CsA/SRL caused the most significant rise in TGF-*β*1 secretion (843.1 ± 7.1 versus 1247.4 ± 10.4 pg/mL; *P* < 0.01). The increase in TGF-*β*1 secretion observed with CsA/SRL was significantly greater than the increase observed with CsA alone (1089.5 ± 26.1 versus 1247.4 ± 10.4 pg/mL; *P* < 0.05).

To further examine the role of TGF-*β*, we decided to investigate the effects of CsA, SRL, and CsA/SRL cotreatment on a Smad-responsive luciferase construct. CsA treatment caused a significant increase in Smad activation (100 versus 156.9 ± 1.7 luciferase units; *P* < 0.05). SRL administration did not significantly alter Smad activation (100 versus 123.5 ± 4.3 luciferase units). Treatment with CsA/SRL cotreatment caused the greatest increase in Smad activation (100 versus 210.1 ± 15.9 luciferase units; *P* < 0.01). The increase in Smad activation in the presence of CsA/SRL cotreatment was significantly greater than with CsA alone (156.9 ± 1.7 versus 210.1 ± 15.9 luciferase units; *P* < 0.01) (Figure 1(c)).

### 3.2. CsA/SRL Cotreatment Increased CTGF and Decreased MMP-9 Gene Expression

**Figure 2:**
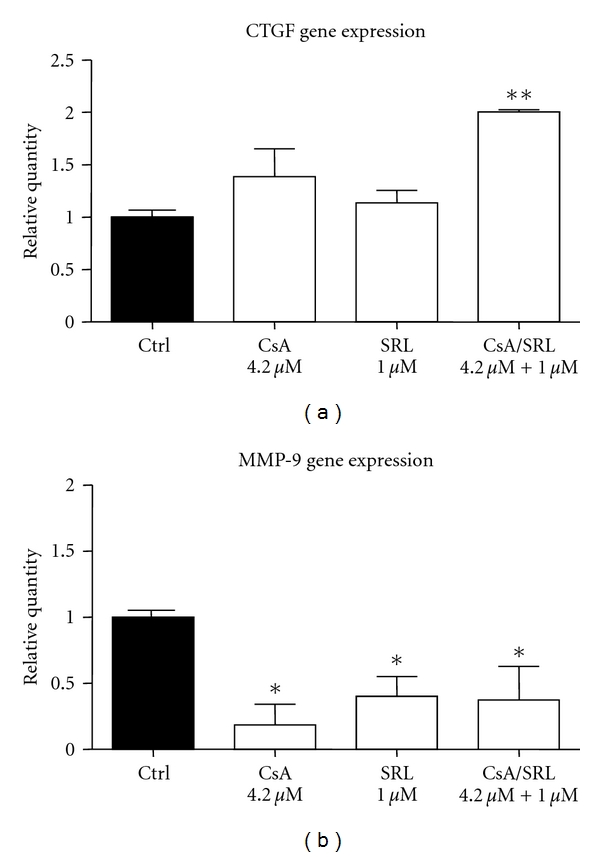
CsA/SRL cotreatment increased CTGF and decreased MMP-9 gene expression in HMCs. HMCs were grown to confluency and treated with vehicle, 4.2 *μ*M CsA, 1 *μ*M SRL or CsA/SRL, 4.2 *μ*M CsA + 1 *μ*M SRL for 24 hours. CTGF and MMP-9 expression was detected by quantitative PCR. (a) 24-hour CTGF gene expression and (b) MMP-9 gene expression. Each column represents the mean ± SEM of a minimum of 3 independent experiments performed in duplicate. Data was analysed by ANOVA and comparisons between control and multiple treatment groups were made using the Dunnet posttest. *Indicates statistical difference compared to control. *P < 0.05, **P < 0.01.

The observation of increased Smad signalling prompted an investigation into a downstream mediator of TGF-*β*, connective tissue growth factor (CTGF). Treatment for 24 hours with CsA showed a trend towards an increase in CTGF gene expression, which was not statistically significant. Treatment with SRL alone caused no significant increase in CTGF gene expression. However, treatment with CsA/SRL cotreatment caused a significant increase in CTGF gene expression (1 versus 2.0 ± 0.02; *P* < 0.01) (Figure 2(a)).

CsA treatment for 24 hours caused a significant decrease in matrix metalloproteinase 9 (MMP-9) gene expression (1.0 versus 0.18 ± 0.15; *P* < 0.05). SRL also caused a significant reduction in MMP-9 gene expression. CsA/SRL cotreatment also caused a significant decrease in MMP-9 gene expression (1 versus 0.37 ± 0.2; *P* < 0.05, Figure 2(b)).

### 3.3. CsA, SRL, and CsA/SRL Increased ERK 1/2 Activity in HMCs

**Figure 3:**
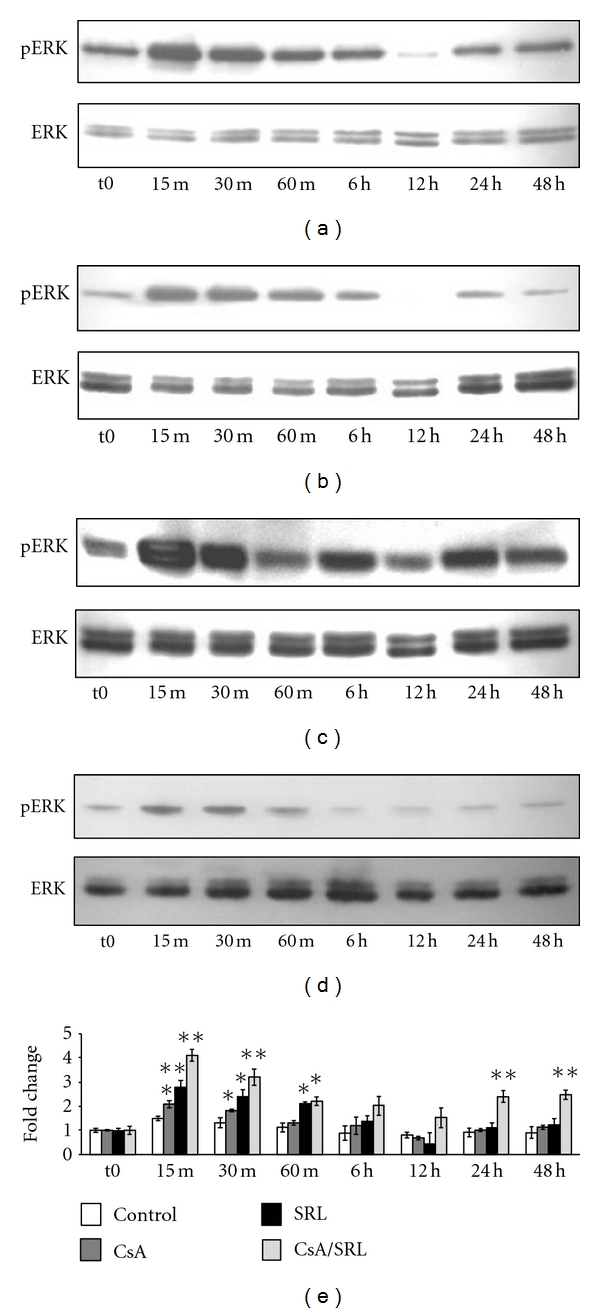
CsA/SRL cotreatment caused a biphasic increase in ERK 1/2 activity in HMCs. HMCs were grown to confluency and treated with vehicle, 4.2 *μ*M CsA, 1 *μ*M SRL, or CsA/SRL 4.2 *μ*M CsA + 1 *μ*M SRL. Phosphorylated ERK 1/2 MAPK or whole-cell ERK 1/2 MAPK was probed by Western blotting. Representative blots from one of three independent experiments are shown. (a) ERK 1/2 phosphorylation following CsA treatment, (b) ERK 1/2 phosphorylation following SRL treatment, (c) ERK 1/2 phosphorylation following CsA/SRL cotreatment. (d) ERK 1/2 phosphorylation in the presence of vehicle (fresh medium was applied to cells at t0). Densitometric analysis was conducted by calculating the ratio of phosphorylated ERK 1/2 compared to total whole-cell ERK 1/2 and then comparing this ratio to time-matched vehicle controls. Each column represents the mean ± SEM of a minimum of 3 independent experiments performed in duplicate. Data was analysed by ANOVA and comparisons between control and multiple treatment groups were made using the Dunnet posttest. *Indicates statistical difference compared to control. *P < 0.05, **P < 0.01.

In order to determine the mechanism of CsA/SRL co-treatment-induced alterations, we investigated the effects of CsA, SRL, and CsA/SRL on ERK 1/2 activation. CsA significantly increased ERK 1/2 phosphorylation after 15, 30, and 60 minutes administration (Figure 3(a)). SRL also significantly increased ERK 1/2 phosphorylation after 15 and 30 and 60 minutes treatment (Figure 3(b)). CsA/SRL co-treatment caused a synergistic increase in ERK 1/2 phosphorylation at 15, 30, and 60 minutes. However, the combination of CsA/SRL co-treatment also significantly increased ERK 1/2 phosphorylation at 24 and 48 hours, unlike the CsA and SRL individual treatments (Figure 3(c)).

### 3.4. Inhibition of ERK 1/2 Provided Partial Protection against CsA/SRL Cytotoxicity

**Figure 4:**
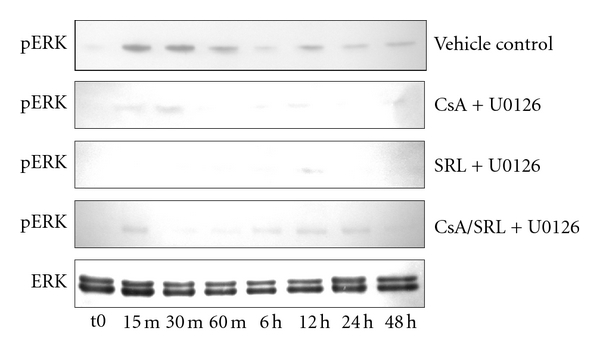
The MEK inhibitor, U0126, blocked CsA- and SRL-induced ERK 1/2 activity in HMCs. HMCs were grown to confluency and treated with vehicle, 4.2 *μ*M CsA, 1 *μ*M SRL, or CsA/SRL 4.2 *μ*M CsA + 1 *μ*M SRL in the presence or absence of 10 *μ*M UO126 for up to 48 hours. Phosphorylated ERK 1/2 MAPK or whole-cell ERK 1/2 MAPK was probed by Western blotting. Representative blots from one of three independent experiments are shown.

**Figure 5:**
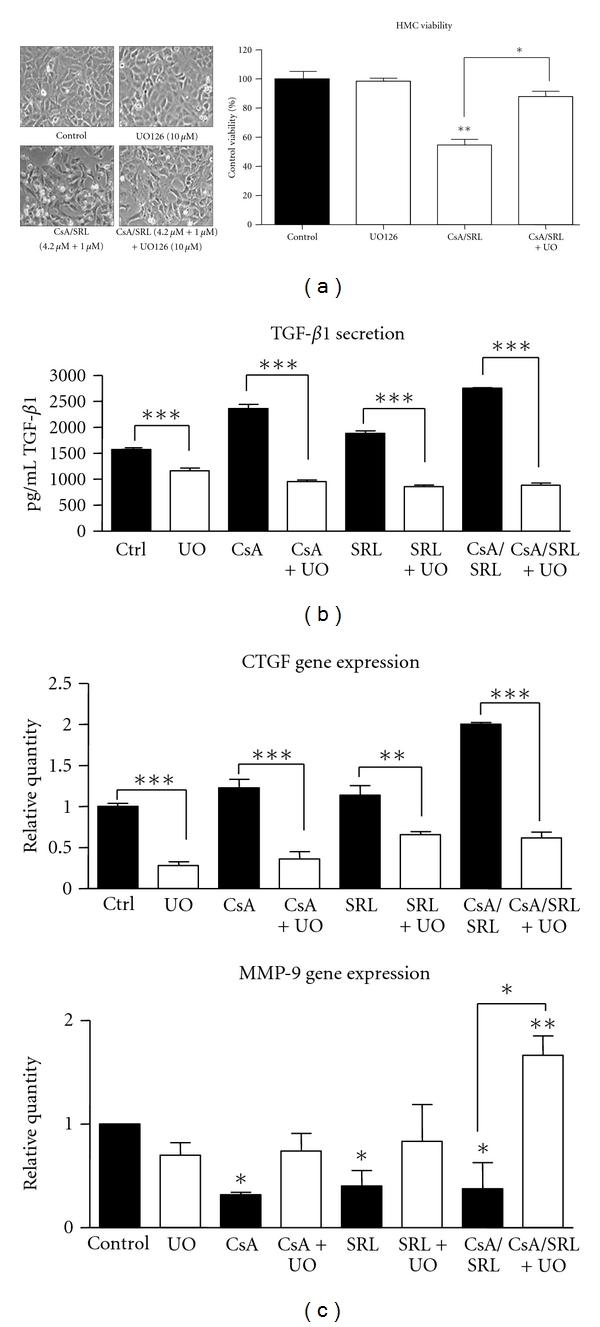
ERK 1/2 inhibition attenuated the CsA/SRL-induced alterations in HMCs. HMCs were grown to confluency and pretreated with UO126 10 *μ*M for 1-hour prior to treatment with vehicle control, 4.2 *μ*M CsA, 1 *μ*M SRL, or CsA/SRL 4.2 *μ*M + 1 *μ*M, for 24 hours. phase contrast micrographs were taken using a CCD camera mounted on a Nikon microscope. (a) Magnification 200X. HMC viability was measured using the Resazurin Conversion Assay. (b) TGF-*β*1 secretion was detected using a TGF-*β*1 ELISA. (c) 24-hour CTGF gene expression and 24-hour MMP-9 gene expression. Gene expression was detected by quantitative PCR. Each column represents the mean ± SEM of a minimum of 3 independent experiments performed in duplicate. Data was analysed by ANOVA and comparisons between control and multiple treatment groups were Mmade using the Dunnet Posttest.*P < 0.05, **P < 0.01, ***P < 0.001.

The addition of UO126 prevented the CsA, SRL, and CsA/SRL co-treatment-induced stimulation of ERK 1/2 (Figure 4). Total levels of ERK 1/2 protein were unaltered following CsA, SRL, or CsA/SRL combination in the presence or absence of UO126 treatment. It was upon the notable activation of ERK 1/2 phosphorylation that the effect of 24-hour CsA/SRL treatment in the presence of the ERK 1/2 inhibitor UO126 was examined (Figure 5). ERK 1/2 inhibition appeared to partially ameliorate the CsA/SRL co-treatment-induced morphological alterations in HMCs. However, there were still some gaps visible in the monolayer. The CsA/SRL-induced reduction in HMC viability was prevented to some extent by ERK inhibition (54 ± 3.9 versus 88 ± 3.5, *P* < 0.001). The addition of the ERK inhibitor alone caused no effect on HMC viability or morphology.

Inhibition of ERK 1/2 phosphorylation prevented the CsA alone and CsA/SRL co-treatment-induced increases in TGF-*β* secretion (Figure 4). (CsA + UO126,  518 ± 23.5  versus 1089 ± 26.1, CsA, *P* < 0.001). (CsA/SRL + UO126,  424 ± 54.2  versus  1247 ± 10.4, CsA/SRL, *P* < 0.001). ERK inhibition also significantly reduced control and SRL levels of TGF-*β*1 secretion.

 ERK inhibition also dramatically reduced the CsA/SRL-induced increase in CTGF gene expression to below control levels (CsA/SRL, 1.7 ± 0.2 versus 0.6 ± 0.14, CsA/SRL + UO126). ERK inhibition also significantly reduced control CTGF gene expression (Figure 4). ERK inhibition also prevented the previously observed CsA/SRL co-treatment-induced decrease in MMP-9 gene expression (0.37 ± 0.25 versus 1.6 ± 0.2; *P* < 0.001) (Figure 4). ERK inhibition also restored the CsA- and SRL-induced decreases in MMP-9 and restored MMP-9 values to control levels. UO126 alone did not significantly alter MMP-9 gene expression. 

## 4. Discussion

The results presented in this body of work demonstrated that the combination treatment of CsA and SRL did not result in an attenuation of the CNI-associated changes in HMCs but in fact resulted in synergistic nephrotoxic-like effects. It appeared that the primary causative factor for the CsA/SRL-induced mesangial cell dysfunction was a significant dual increase in TGF-*β* and CTGF expression. We also observed enhanced activation of the ERK 1/2 MAPK pathway, the TGF-*β* signalling pathway, and alteration of ECM regulators.

 Altered HMC morphology and viability gave the first indication that CsA and SRL combination treatment was exerting a detrimental effect when administered to HMCs in the present study. These initial findings of enhanced toxicity were surprising as it had been previously reported that SRL had a protective role and could reduce the toxicity observed with CNI administration [22, 23]. However, more recently, experimental evidence has emerged suggesting that a combination of CsA and SRL might not be protective. In a rat model, kidney function and morphology were assessed following short-term combination therapy with CsA, and SRL [9]. Striped fibrosis and glomerular filtration rate (GFR) were significantly worse in rats receiving CsA and SRL combination according to the study by Nielsen et al. [9].

 We observed that the secretion of TGF-*β* was significantly increased following immunosuppressive drug treatment in the present model. TGF-*β* is a major factor involved in CsA-induced renal fibrosis and renal disease [24]. TGF-*β* has been shown to be upregulated in a number of *in vitro* and *in vivo *experimental models following CsA treatment and in patients undergoing CsA therapy [14, 15, 25]. Increased TGF-*β* may have contributed to the adverse effects observed in the present study. The lack of an increase in TGF-*β* as observed with SRL alone in the current study may help to explain the reduced nephrotoxic side effects associated with SRL [4].

 It is accepted now that altered pharmacokinetics have a role in contributing to the observed toxicity with a combination of SRL and CsA [26]. An *in vitro *study in human renal epithelial cells demonstrated that SRL prolonged the intracellular accumulation of CsA when given in combination. The authors showed that SRL inhibited P-glycoprotein-mediated efflux contributing to CsA nephrotoxicity [27]. However, the reasons for the augmented nephrotoxicity resulting from a combination of SRL and CsA still remain to be fully defined, with some studies attributing the effect to pharmacokinetics and others to hyperglycaemia [10, 26, 27]. Another known contributor to CsA nephrotoxicity is TGF-*β*.

 In the present HMC model, SRL had a synergistic effect on TGF-*β* secretion when coadministered with CsA. However, similar increases in TGF-*β* were also reported in a study on CsA and SRL combination therapy by Shihab et al. [11]. In that study, rats were given doses of CsA alone, SRL alone, or a combination of CsA and SRL. The authors observed a worsening in renal function in the group that were given CsA and SRL in combination compared to CsA and SRL alone. The CsA/SRL group also exhibited tubular injury, interstitial fibrosis, and arteriolopathy as indicated by histological analysis. Elevated TGF-*β* protein levels were also observed in the CsA and SRL co-treatment groups, and the levels were significantly higher than with CsA or SRL alone [11]. To our knowledge, however, this is the first time that this effect has been observed in HMCs.

 One downstream mediator of TGF-*β* is CTGF. CTGF was upregulated in the present HMC model following CsA/SRL co-treatment. CTGF has been found to be upregulated in many inflammatory glomerular diseases and in patient biopsies with numerous different conditions including glomerulonephritis and focal segmental glomerulosclerosis [28]. It appeared that a novel dual elevation of TGF-*β* and CTGF in the HMCs following CsA/SRL co-treatment contributed to the enhanced CsA/SRL-induced mesangial cell alterations. This dual elevation of TGF-*β* and CTGF was not observed following treatment with CsA or SRL alone.

 In the current study, CsA/SRL may also have altered ECM turnover by decreasing MMP-9 levels. The observed reduction in MMP-9 in the present mesangial cell model may have facilitated ECM accumulation by preventing ECM degradation. It is known that ECM regulators are extremely context specific and chronologically variable [29]. In models of chronic kidney disease, decreased MMP-9 has been shown to aid disease progression. In a rat ischemia-reperfusion model, Caron et al. observed that MMP-9 was significantly upregulated and may have contributed to glomerular injury [30]. In another model, transgenic mice overexpressing renin developed hypertension-induced renal dysfunction. Isolated glomeruli from these mice exhibited elevated TGF-*β* accompanied with decreased MMP-9 gene expression [31].

 These alterations in HMCs following CsA/SRL treatment are at least in part due to enhanced signalling through the ERK 1/2 pathway. Previous studies in our laboratory have indicated that the MAPK pathways are also activated in response to CsA in Madin Darby canine kidney (MDCK) epithelial cells [32, 33]. A biphasic activation of ERK 1/2 following CsA/SRL co-treatment was observed in the HMCs and may be the mechanism by which the synergistic nephrotoxic effect is exerted by the CsA/SRL co-treatment. This is further supported by the fact that ERK 1/2 inhibition resulted in significant attenuation of this CsA/SRL-induced HMC dysfunction. Considering the well-characterised roles of ERK 1/2 signalling in cell survival and proliferation, our finding that enhanced ERK 1/2 signalling is central to CsA/SRL cytotoxicity is interesting. These observations are in keeping with other studies in HMCs. Ishikawa and Kitamura observed that incubation of cultured HMCs with hydrogen peroxide induced apoptosis [34]. This apoptosis was accompanied by activation of the ERK 1/2 MAPK pathway. Pharmacological inhibition of ERK 1/2 attenuated the hydrogen peroxide-induced mesangial cell apoptosis [34]. Similarly, in cisplatin-induced cytotoxicity, ERK 1/2 has been shown to play a central role [35]. Several mechanisms of ERK 1/2-mediated cytotoxicity have been proposed including ERK-dependent activation of caspases [36] and induction of autophagy [37]. Importantly from the perspective of this study, in examples of ERK-mediated cytotoxicity, ERK activation is unusually prolonged (up to 72 hours) [38]. Therefore, the prolonged ERK activation observed in this study, in the presence of CsA/SRL co-treatment, may be a key mechanistic difference compared to the two immunosuppressants in isolation. Blockade of the ERK 1/2 pathway also caused significant attenuation of TGF-*β* secretion. This suggests that the ERK 1/2 pathway may play a major role in TGF-*β* signalling. ERK 1/2 also appeared to play a role in CTGF expression in this mesangial cell model as CTGF gene expression was attenuated by ERK inhibition. These observations have been observed in other models of CsA nephrotoxicity [39, 40]. 

Our proposed mechanism for the enhanced CsA/SRL nephrotoxicity is that CsA/SRL strongly activates ERK 1/2, which promotes TGF-*β* cellular secretion. The excess TGF-*β* then promotes increased CTGF via the Smad pathway. TGF-*β* may also promote decreased ECM degradation via decreased MMP9, which contributes to the observed mesangial cell dysfunction. These findings are important in determining future immunosuppression strategies for SRL therapy in clinical organ transplantation. Currently, there are two main strategies: *de novo *use of SRL in combination with reduced amounts of CsA or complete conversion from a CNI-based protocol to SRL in cases where well-recognized adverse effects of CNIs (such as impaired renal function) are prevalent [33]. However, there is much debate on this issue and longer-term studies in larger cohorts of patients are required to determine whether replacement of CsA with SRL provides any significant improvement in patient and graft survival [33]. The results shown in this study would suggest that long-term use of SRL in patients will have serious nephrotoxic effects.

##  Funding

This work was supported by the Health Research Board, Science Foundation Ireland, Enterprise Ireland, and the programme for research in third level institutions administered by the Higher Education Authority and by the EU 7th Framework Grant “SysKid”, HEALTH-F2-2009-241544. C. Slattery is funded by a Government of Ireland Research Fellowship from the Irish Research Council for Science, Engineering and Technology.

##  Conflict of Interests

The authors have no conflict of interest. The results presented in this paper have not been published previously in whole or part, except in abstract form.
